# Google Trends in Infodemiology and Infoveillance: Methodology Framework

**DOI:** 10.2196/13439

**Published:** 2019-05-29

**Authors:** Amaryllis Mavragani, Gabriela Ochoa

**Affiliations:** 1 Department of Computing Science and Mathematics Faculty of Natural Sciences University of Stirling Stirling United Kingdom

**Keywords:** big data, health, infodemiology, infoveillance, internet behavior, Google Trends

## Abstract

Internet data are being increasingly integrated into health informatics research and are becoming a useful tool for exploring human behavior. The most popular tool for examining online behavior is Google Trends, an open tool that provides information on trends and the variations of online interest in selected keywords and topics over time. Online search traffic data from Google have been shown to be useful in analyzing human behavior toward health topics and in predicting disease occurrence and outbreaks. Despite the large number of Google Trends studies during the last decade, the literature on the subject lacks a specific methodology framework. This article aims at providing an overview of the tool and data and at presenting the first methodology framework in using Google Trends in infodemiology and infoveillance, including the main factors that need to be taken into account for a strong methodology base. We provide a step-by-step guide for the methodology that needs to be followed when using Google Trends and the essential aspects required for valid results in this line of research. At first, an overview of the tool and the data are presented, followed by an analysis of the key methodological points for ensuring the validity of the results, which include selecting the appropriate keyword(s), region(s), period, and category. Overall, this article presents and analyzes the key points that need to be considered to achieve a strong methodological basis for using Google Trends data, which is crucial for ensuring the value and validity of the results, as the analysis of online queries is extensively integrated in health research in the big data era.

## Introduction

The use of internet data has become an integral part of health informatics over the past decade, with online sources becoming increasingly available and providing data that can be useful in analyzing and predicting human behavior. This use of the internet has formed two new concepts: “Infodemiology,” first defined by Eysenbach as “the science of distribution and determinants of information in an electronic medium, specifically the Internet, or in a population, with the ultimate aim to inform public health and public policy” [1], and “Infoveillance,” defined as “the longitudinal tracking of infodemiology metrics for surveillance and trend analysis” [2].

The main limitation of validating this line of research is the general lack of openness and availability of official health data. Data collection and analysis of official health data on disease occurrence and prevalence involve several health officials and can even take years until the relevant data are available. This means that data cannot be accessed in real time, which is crucial in health assessment. In several countries, official health data are not publicly available, and even in countries where data are available, they usually consist of large time-interval data (eg, annual data), which makes the analysis and forecasting of diseases and outbreaks more difficult.

Nevertheless, data from several online sources are being widely used to monitor disease outbreaks and occurrence, mainly from Google [3-7] and social media [8-12]. Twitter has become increasingly popular over the past few years [13-19], while several other studies have combined data from different online sources such as Facebook and Twitter [20] or Google, Twitter, and electronic health records [21].

Currently, the most popular tool in addressing health issues and topics with the use of internet data is Google Trends [22], an open online tool that provides both real-time and archived information on Google queries from 2004 on. The main advantage of Google Trends is that it uses the revealed and not stated users’ preferences [23]; therefore, we can obtain information that would be otherwise difficult or impossible to collect. In addition, as data are available in real time, it solves issues that arise with traditional, time-consuming survey methods. Another advantage is that, as Web searches are performed anonymously, it enables the analysis and forecasting of sensitive diseases and topics, such as AIDS [24], mental illnesses and suicide [25-27], and illegal drugs [28,29].

Despite the limitations of data from traditional sources and owing to the fact that online data have shown to be valuable in predictions, the combination of traditional data and Web-based data should be explored, as the results could provide valid and interesting results. Over the past few years, the diversity of online sources used in addressing infodemiology topics is increasing. Indicative recent publications of online sources and combinations of sources are presented in Table 1.

As discussed above, many studies have used Google Trends data to analyze online behavior toward health topics and to forecast prevalence of diseases. However, the literature lacks a methodology framework that provides a concise overview and detailed guidance for future researchers. We believe such a framework is imperative, as the analysis of online data is based on empirical relationships, and thus, a solid methodological basis of any Google Trends study is crucial for ensuring the value and validity of the results.

**Table 1:** Recent indicative infodemiology studies.

Author(s)	Keywords	Google Trends	Twitter	Facebook	Other social media (eg, YouTube)	Blogs, forums, news outlets, Wikipedia	Databases, electronic health records	Other search engines (Baidu)
Abdellaoui et al [30]	Drug treatment					✓		
Allen et al [31]	Tobacco waterpipe		✓					
Berlinger et al [32]	Herpes, Vaccination	✓						
Bragazzi and Mahroum [33]	Plague, Madagascar	✓						
Chen et al [18]	Zika epidemic		✓					
Forounghi et al [34]	Cancer	✓						
Gianfredi et al [35]	Pertussis	✓						
Hswen et al [36]	Psychological analysis, Autism		✓					
Jones et al [37]	Cancer					✓		
Kandula et al [38]	Influenza	✓						
Keller et al [39]	Bowel disease, Pregnancy, Medication			✓	✓	✓		
Mavragani et al [7]	Asthma	✓						
Mejova et al [40]	Health monitoring			✓				
Odlum et al [41]	HIV/AIDS		✓					
Phillips et al [42]	Cancer	✓						
Poirier et al [43]	Influenza, Hospitals	✓					✓	
Radin et al [44]	Systematic Lupus Erythematous	✓						
Roccetti et al [20]	Crohn’s disease		✓	✓				
Tana et al [25]	Depression, Finland	✓						
Vsconcellos-Silva et al [45]	Cancer	✓						
Wakamiya et al [46]	Influenza		✓					
Wang et al [47]	Obesity	✓						
Watad et al [48]	West Nile Virus	✓			✓	✓		
Xu et al [49]	Cancer, China							✓

We proceed in a step-by-step manner to develop the methodology framework that should be followed when using Google Trends in infodemiology. First, we provide an overview of how the data are retrieved and adjusted along with the available features, followed by the methodology framework for choosing the appropriate keyword(s), region(s), period, and category. Finally, the results are discussed, along with the limitations of the tool and suggestions for future research.

## Methodology Framework

### Data Overview

Google Trends is an open online tool that provides information on what was and is trending, based on actual users’ Google queries. It offers a variety of choices, such as Trending Searches, Year in Search, and Explore. Table 2 describes the features offered by Google Trends and their respective descriptions.

When using Google Trends for research, data are retrieved from the “Explore” feature, which allows download of real-time data from the last week and archived data for specific keywords and topics from January 2004 up to 36 hours before the search is conducted. The data are retrieved directly from the Google Trends Explore page in .csv format after the examined keyword(s) is entered and the region, period, and category are selected. By default, the period is set to “Worldwide,” the time frame is set to “past 12 months,” and the category is set to “All categories.”

The data are normalized over the selected time frame, and the adjustment is reported by Google as follows:

Search results are proportionate to the time and location of a query by the following process: Each data point is divided by the total searches of the geography and time range it represents to compare relative popularity. Otherwise, places with the most search volume would always be ranked highest. The resulting numbers are then scaled on a range of 0 to 100 based on a topic’s proportion to all searches on all topics. Different regions that show the same search interest for a term don't always have the same total search volumes [50]

The normalization of data indicates that the values vary from 0 to 100. The value 0 does not necessarily indicate no searches, but rather indicates very low search volumes that are not included in the results. The adjustment process also excludes queries that are made over a short time frame from the same internet protocol address and queries that contain special characters. Google does not have a filter for controversial topics, but it excludes related search terms that are sexual. However, it allows retrieval of queries’ normalized hits for any keyword entered, independent of filters.

**Figure 1:**
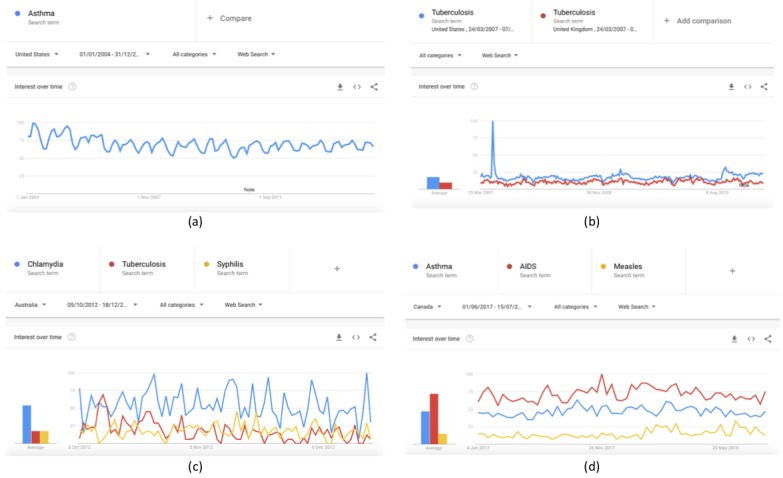
Graphs of the variations in the online interest for the examined terms over the selected time frame in Google Trends.

Google Trends allows one to explore the online interest in one term or the comparison of the online interest for up to five terms. It allows a variety of combinations to compare different terms and regions as follows:

- For one term in one region over a specific period, such as for “Asthma” in the United States from January 2004 to December 2014 (Figure 1a)
- For the same term in different regions over the same period, such as for “Tuberculosis” in the United States and United Kingdom from March 24, 2007, to April 7, 2011 (Figure 1b)
- For different terms (up to five) in the same region for the same period, such as for the terms “Chlamydia,” “Tuberculosis,” and “Syphilis” in Australia from October 5, 2012, to December 18, 2012 (Figure 1c)
- For different terms (up to five) for different regions over the same period, such as comparing the term “Asthma” in the United States, “AIDS” in the United Kingdom, and “Measles” in Canada from June 1, 2017, to July 15, 2018 (Figure 1d)

**Figure 2:**
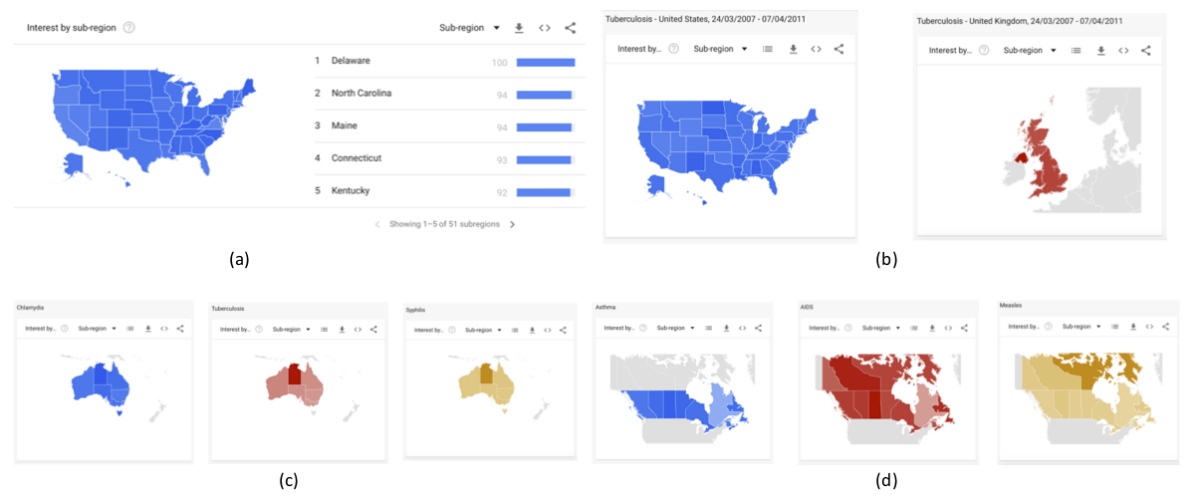
Heat map for (a) “Asthma” in the United States from Jan 2004 to Dec 2014; (b) “Tuberculosis” in the United States and United Kingdom from March 24, 2007, to April 7, 2011; (c) “Chlamydia,” “Tuberculosis,” and “Syphilis” in Australia from Oct 5, 2012, to Dec 18, 2012; (d) “Asthma” in the United States, “AIDS” in the United Kingdom, and “Measles” in Canada from June 1, 2017, to July 15, 2018.

When the term(s), region(s), period(s), and category are defined, the outputs are a graph of the variations of all examined terms in the online interest over the selected time frame (Figure 1) and their respective heat maps, which are presented separately for all examined regions (Figure 2); all datasets can be downloaded in .csv format.

**Figure 3:**
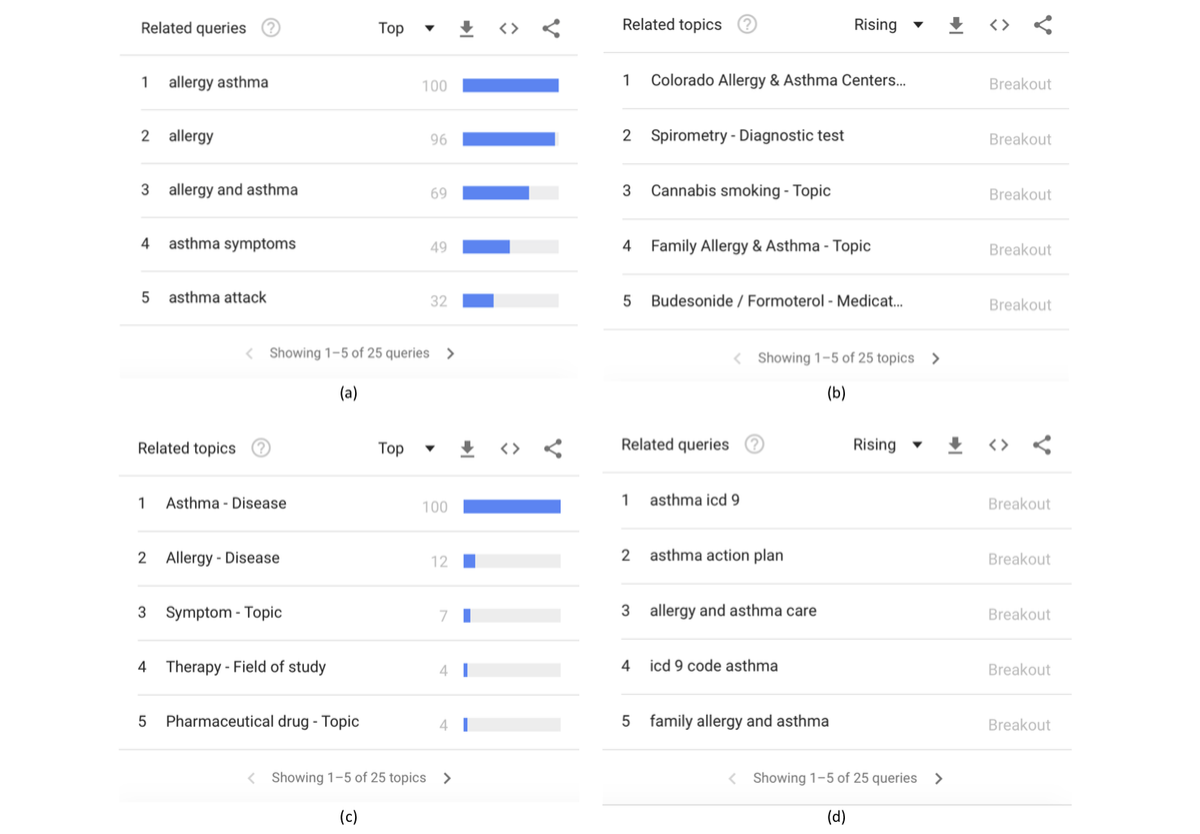
Google Trends’ (a) top related queries, (b) rising related topics, (c) top related topics, and (d) rising related queries for “Asthma” in the United States from Jan 1, 2004, to Dec 31, 2014.

Apart from the graph, the .csv with the relative search volumes, and the interest heat maps, Google Trends also shows and allows one to download .csv files of (1) the “Top related queries”, defined as “Top searches are terms that are most frequently searched with the term you entered in the same search session, within the chosen category, country, or region” (Figure 3a); (2) the “Rising related queries”, defined as "terms that were searched for with the keyword you entered...which had the most significant growth in volume in the requested time period” (Figure 3b); (3) the “Top Related Topics” (Figure 3c); and (4) the “Rising Related Topics” (Figure 3d).

**Table 2:** Google Trends Features and Descriptions.

Feature	Description
Homepage	Provides an overview of what is searched for in a selected region (default: United States)
Explore	Allows exploration of the online interest for specific keywords over selected periods and regions (default: worldwide, 12 months)
Trending Searches	Shows the trending queries for (1) daily search trends and (2) real-time search trends in a selected region (default: United States)
Year in Searches	Show what was trending in a specific region in a specific year (default: United States, previous year)
Subscriptions	Allows subscription for (1) a specific topic in a specific region and sends updates for noteworthy events (via email either once a week or once a month) and (2) trending searches and sends updates about trending searches (via email either as it happens, or once a day, or once a week and includes either “Top Daily Searches,” “Majority of Daily Search Trends,” or “All Daily Search Trends”)

### Keyword Selection

The selection of the correct keyword(s) when examining online queries is key for valid results [51]. Thus, many factors should be taken into consideration when using Google Trends data in order to ensure a valid analysis.

Google Trends is not case sensitive, but it takes into account accents, plural or singular forms, and spelling mistakes. Therefore, whatever the choice of keywords or combination of keywords, parts of the respective queries will not be considered for further analysis.

**Figure 4:**
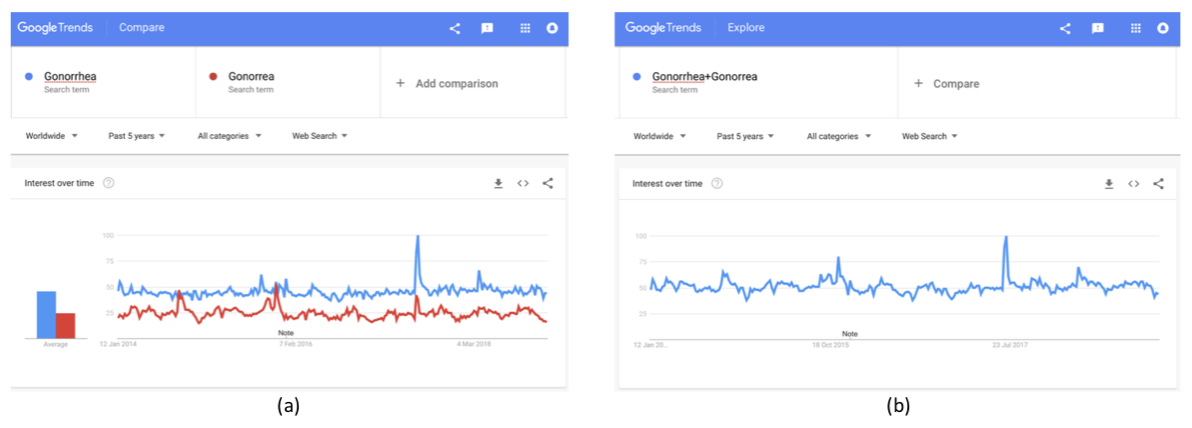
Use of the “+” feature for including misspelled terms for (a) "Gonorrhea" compared to "Gonorrea"; (b) both terms by using the “+” feature.

To partly overcome this limitation, the “+” feature can be used to include the most commonly encountered misspellings, which are selected and entered manually; however, we should keep in mind that some results will always be missing, as all possible spelling variations cannot be included. In addition, incorrect spellings of some words could be used even more often than the correct one, in which case, the analysis will not be trivial. However, in most of the cases, the correct spelling is the most commonly used, and therefore, the analysis can proceed as usual. For example, gonorrhea is often misspelled, mainly as “Gonorrea,” which is also the Spanish term for the disease. As depicted in Figure 4a, both terms have significantly high volumes. Therefore, to include more results, both terms could be entered as the search term by using the “+” feature (Figure 4b). In this way, all results including the correct and the incorrect spellings are aggregated in the results. Note that this is not limited to only two terms; the “+” feature can be used for multiple keywords or for results in multiple languages in a region.

**Figure 5:**
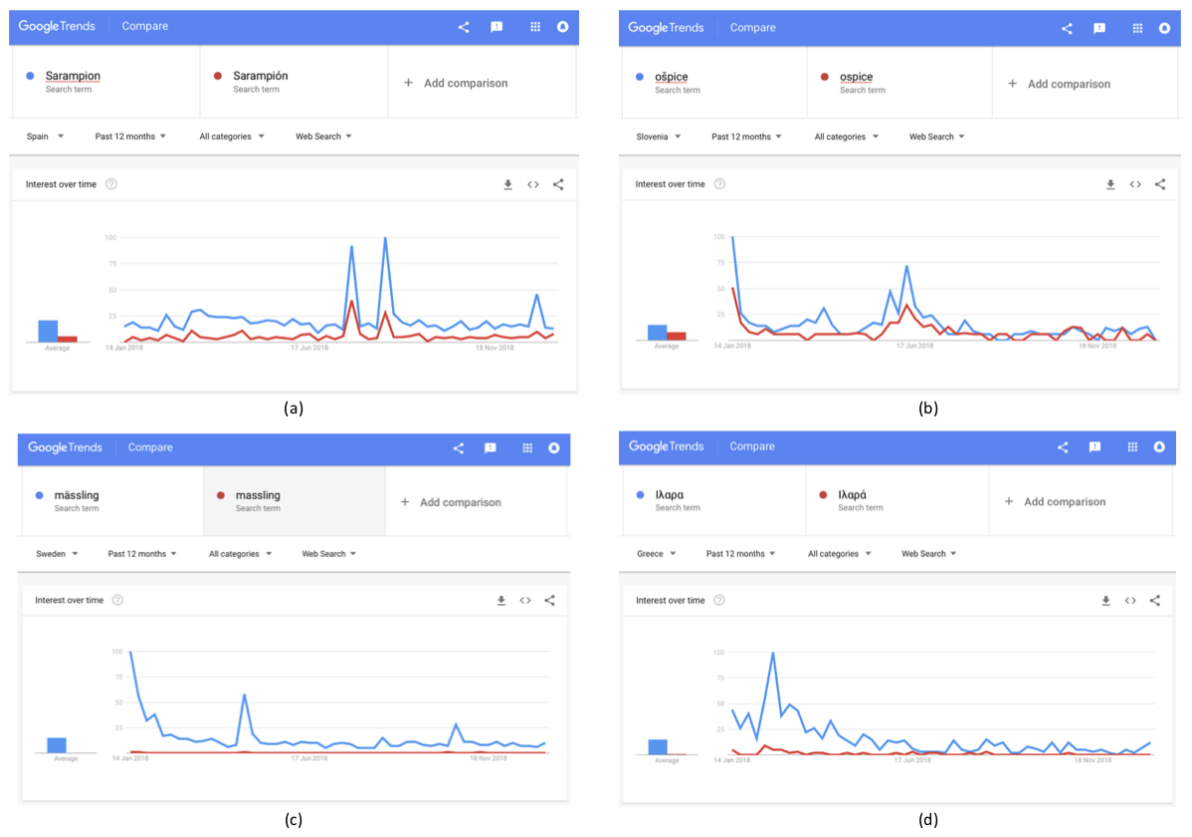
Selection of the correct keyword for measles based on the use of accents in the respective translated terms in (a) Spanish, (b) Slovenian, (c) Swedish, and (d) Greek.

In the case of accents, before choosing the keywords to be examined, the variations in interest between the terms with and those without accents and special characters should be explored. For example, measles translates into “Sarampión,” “ošpice,” “mässling,” and “Ιλαρά” in Spanish, Slovenian, Swedish, and Greek, respectively. As depicted in Figure 5, in Spanish and Greek, the term without the accent is searched for in higher volumes; in Slovenian, the term with the accent is mostly used; and in Swedish, the term without the accent is almost nonexistent. Thus, in Greek searches, the term without accent should be selected, in Slovenian and Swedish searches, terms with accents should be used, while for Spanish, as both terms yield significant results, either both terms using the “+” feature or the term without the accent should be selected.

**Figure 6:**
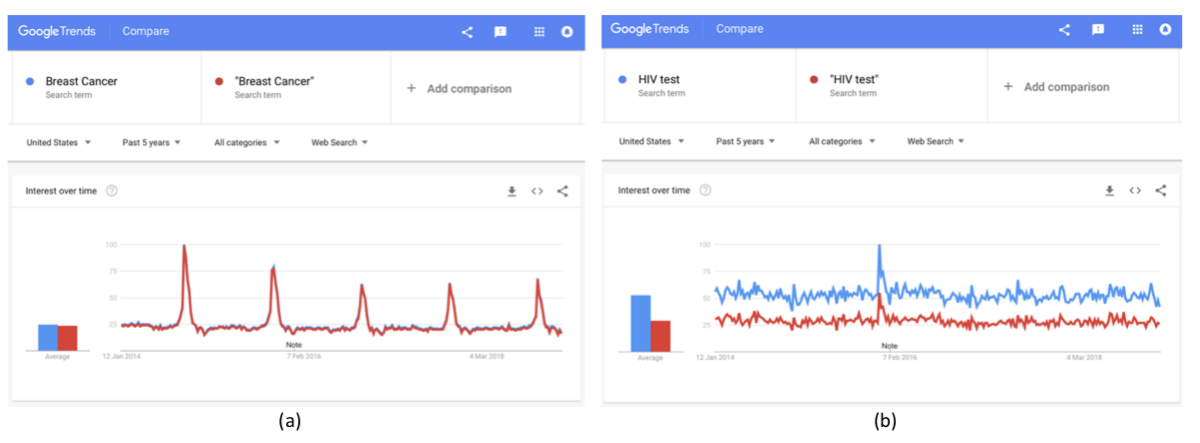
Differences in results with and without quotation marks for (a) “Breast Cancer” and (b) “HIV test.”.

Another important aspect is the use of quotation marks when selecting the keyword. This obviously applies only to keywords with two or more words. For example, breast cancer can be searched online by using or not using quotes. To elaborate, the term “breast cancer” without quotes will yield results that include the words “breast” and “cancer” in any possible combination and order; for example, keywords “breast cancer screening” and “breast and colon cancer” are both included in the results. However, when using quotes, the term “breast cancer” is included as is; for example, “breast cancer screening,” “living with breast cancer,” and “breast cancer patient.” As shown in Figure 6a, the results are almost identical in this case. However, this is not always the case. As depicted in Figure 6b, this is clearly different for “HIV test.” When searching for HIV test with and without quotes, the results differ in volumes of searches, despite the trend being very similar but not exactly the same.

Finally, when researching with Google Trends, the options of “search term” and “disease” (or “topic”) are available when entering a keyword. Although the “search term” gives results for all keywords that include the selected term, “disease” includes various keywords that fall within the category, or, as Google describes it, “topics are a group of terms that share the same concept in any language.”

Therefore, it is imperative that keyword selection is conducted with caution and that the available options and features are carefully explored and analyzed. This will ensure validity of the results.

### Region Selection

The next step is to select the geographical region for which query data are retrieved. The first level of categorization allows data download for the online interest of one or more terms worldwide or by country. The list available includes all countries, in most of which interest in smaller regions can be explored.

**Figure 7:**
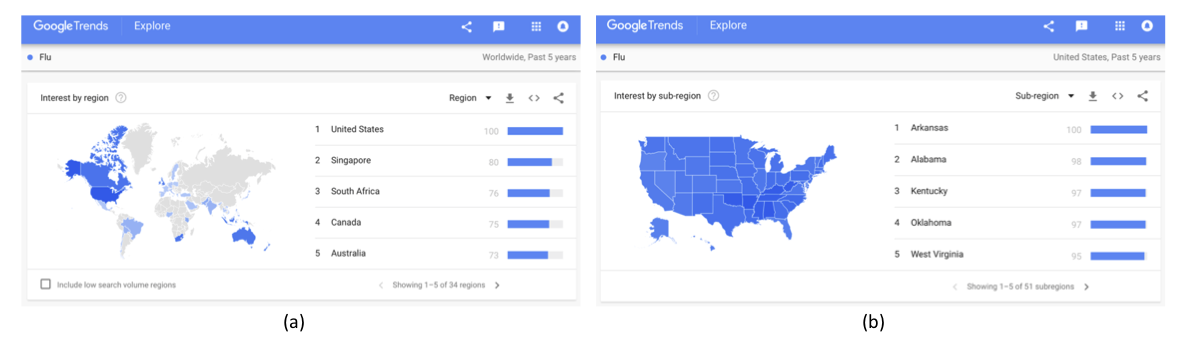
Online interest in the term “Flu” over the past 5 years (a) worldwide and (b) in the United States.

For example, in the United States, it is possible to compare results even at metropolitan and city levels. Figure 7a shows the regional online interest in the term “Flu” worldwide, where the United States is the country with the highest online interest in the examined term, followed by the rest of the 33 countries in which the examined term is most popular. Figure 7b shows the heat map of the interest by state in the United States in the term “Flu” over the past 5 years; either as a new independent search or by clicking on the country “USA” in the worldwide map. As shown in the right bottom corner of Figure 7, Google Trends provides the relative interest for all 50 US states plus Washington DC.

**Figure 8:**
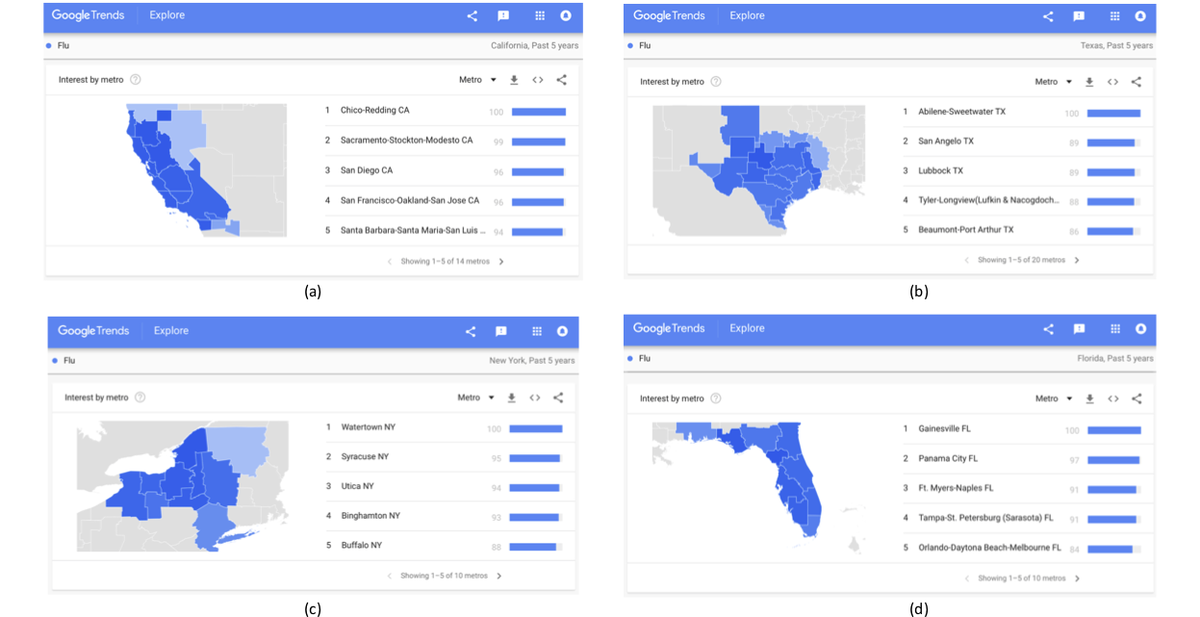
Regional online interest in the term “Flu” at metropolitan level over the past 5 years in (a) California, (b) Texas, (c) New York, and (d) Florida.

**Figure 9:**
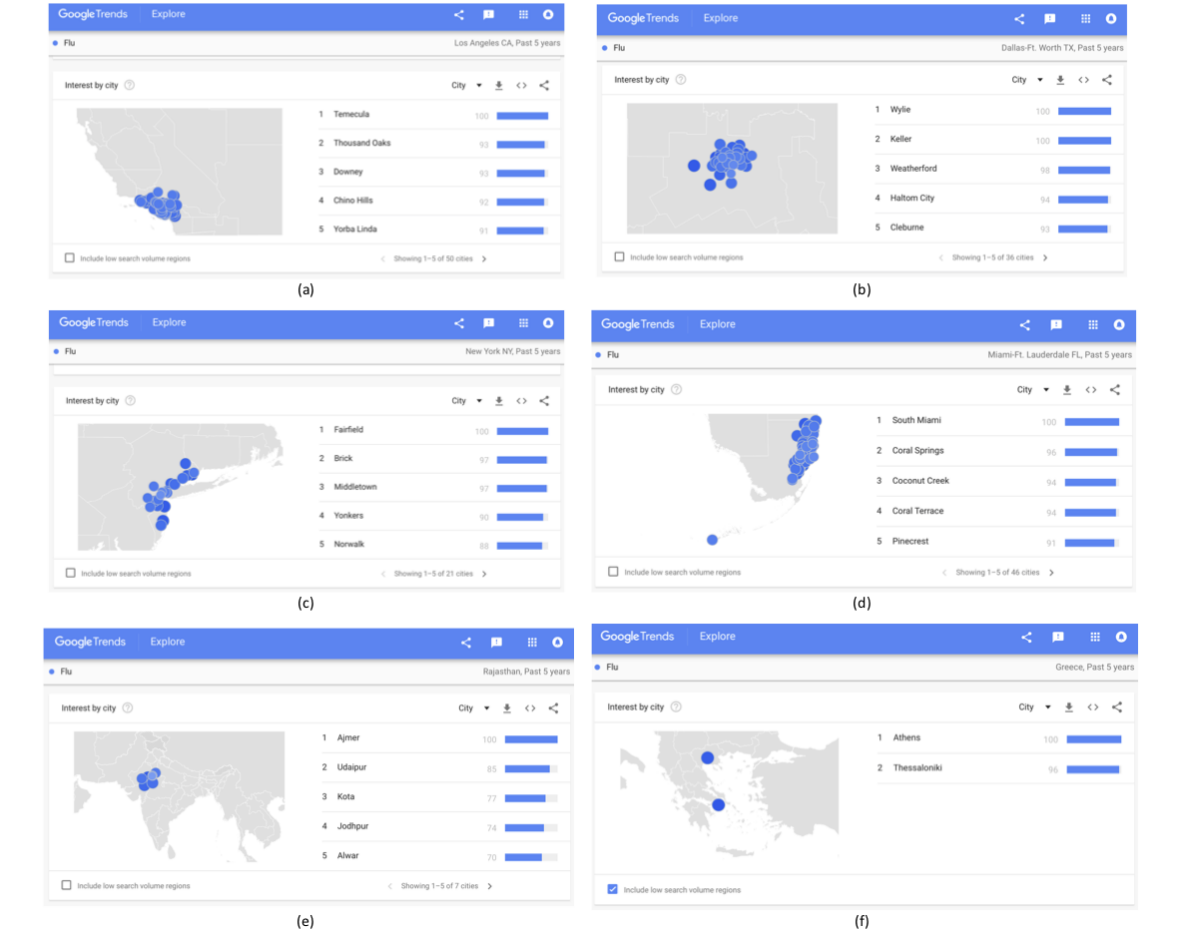
Regional online interest in the term “Flu” at city level over the past 5 years in (a) Los Angeles, (b) Dallas, (c) New York, (d) Miami, (e) India, and (f) Greece.

In the case of the United States, it is possible to examine the online interest by metropolitan area, as depicted in Figure 8 with the examples of California, Texas, New York, and Florida. The option for examining the online interest at the metropolitan level is not available for all countries, where from the state (or county) level, the interest changes directly to the city level. This includes fewer cities than regions with available metropolitan area data, as, for example, in countries with very large populations like India (Figure 9e) or with smaller populations like Greece (Figure 9f).

Figure 9 depicts the online interest by city in the selected metropolitan areas of Los Angeles in California, Dallas in Texas, New York in New York, and Miami in Florida.

At metropolitan level, by selecting the “include low search volume regions,” the total of the included cities is 123 in Los Angeles, 67 in Texas, 110 New York, and 50 in Miami, while in India and Greece, the number of cities remains 7 and 2, respectively.

### Period Selection

As the data are normalized over the selected period, the time frame for which Google Trends data are retrieved is crucial for the validity of the results. The selection of the examined time frame is one of the most common mistakes in Google Trends research. The main guideline is that the period selected for Google data should be exactly the same as the one for which official data are available and will be examined. For example, if monthly (or yearly) official data from January 2004 to December 2014 are available, then the selected period for retrieving Google Trends data should be January 2004 to December 2014. Neither 15 datasets for each individual year nor a random number of datasets arbitrarily chosen should be used; a single dataset should be compiled including the months from January 2004 to December 2014. Note that data may slightly vary depending on the time of retrieval; thus, the date and time of downloading must be reported.

Depending on the time frame, the interval for which data are available varies significantly (Table 3), which includes the data intervals for the preselected time frames in Google Trends. Note that the default selection is 12 months.

**Figure 10:**
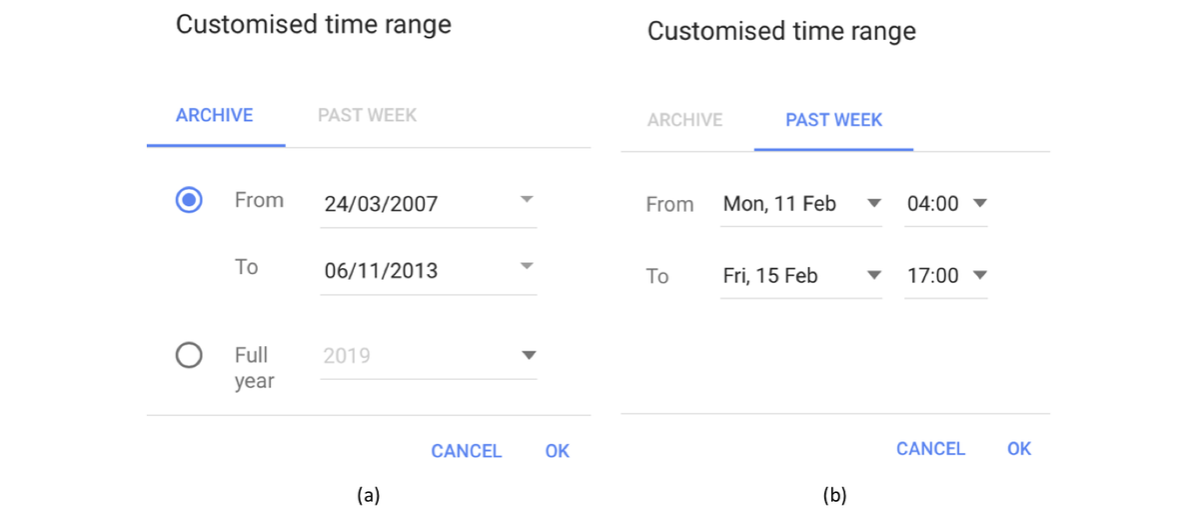
Customized time range (a) from archive and (b) over the past week.

The time frame can be customized at will; for example, March 24, 2007, to November 6, 2013 (Figure 10 a). Furthermore, there is an option to select the exact hours for which data are retrieved, but only over the past week; for example, from February 11, 4 am, to February 15, 5 pm (Figure 10 b).

Finally, an important detail in the selection of the time frame is when the data retrieval changes from monthly to weekly and weekly to daily. For example, from April 28, 2013, to June 30, 2018, the data are retrieved in weekly intervals, while from April 27, 2013, to June 30, 2018, the data are retrieved in monthly intervals. Hence, the data from monthly to weekly changes in (roughly) 5 years and 2 months. For daily data, we observe that, for example, from October 4, 2017, to June 30, 2018, the data are retrieved in daily intervals, while from October 3, 2017, to June 30, 2018, the data are retrieved in weekly intervals; as such, the data interval changes from daily to weekly in (roughly) 10 months.

**Table 3:** Data intervals and number of observations for the default options in period selection.

Selected period	Data intervals	Number of observations
2004 to present	Monthly	>187
Past 5 years	Weekly	260
Full year (eg, 2004 or 2008)	Weekly	52
Past 12 months	Weekly	52
Past 90 days	Daily	90
Past 30 days	Daily	30
Past 7 days	Hourly	168
Past day	8 min	180
Past 4 hours	1 min	240
Past hour	1 min	60

### Search Categories

When exploring the online interest, the selected term can be analyzed based on a selected category. This feature is important to eliminate noisy data, especially in cases where the same word is used or can be attributed to different meanings or events. For example, the terms “yes” and “no” are very commonly searched for, so, when aiming at predicting the results of a referendum race, the search must be limited to the category “Politics” or “Campaign and elections” in order to retrieve the data that are attributed to the event. However, selecting a category is not required when the keyword searched is specific and not related to other words, meanings, and events.

The available categories are listed in Table A1 of Multimedia Appendix 1. Note that most of these categories have subcategories, which, in turn, have other subcategories, allowing the available categories to be as broad or as narrow as required.

In this paper, we focus on the category of “Health” (first level of categorization). The main available subcategories (second level of categorization) of “Health” along with all available subcategories (third and fourth levels) are presented in Table A2 of Multimedia Appendix 1.

Finally, another feature is the type of search conducted when entering a keyword, which consists of the options of “Web Search,” “Image Search,” “News Search,”“Google Shopping,” and “YouTube Search.” Apart from very specific cases, the “Web Search,” which is also the default option, should be selected.

## Discussion

Over the past decade, Web-based data are used extensively in digital epidemiology, with online sources playing a central role in health informatics [1,2,52]. Digital disease detection [53] consists of detecting, analyzing, and predicting disease occurrence and spread, and several types of online sources are used, including mainly digital platforms [54,55]. When addressing infodemiology topics, a concept first introduced by Eysenbach [1], Google Trends is an important tool, and research on the subject is constantly expanding [56]. Most studies on Google Trends research are in health and medicine, focusing mainly on the surveillance and analysis of health topics and the forecasting of diseases, outbreaks, and epidemics. As Google Trends is open and user friendly, it is accessed and used by several researchers, even those who are not strictly related to the field of big data, but use it as a means of exploring behavioral variations toward selected topics. The latter has resulted in differences in methodologies followed, which, at times, involve mistakes.

Despite the large number of studies in this line of research, there was a lack of a methodology framework that should be followed. This has produced differences in presentation, and, more importantly, in crucial mistakes that compromise the validity of the results. In this article, we provided a concise overview of the how the tool works and proposed a step-by-step methodology (ie, the four steps of selecting the correct/appropriate keyword, region, period, and category) to ensure the validity of the results in Google Trends research. We also included research examples to provide guidance not only to the experienced eye, but also to new researchers.

As is evident by the findings of this study, there are several limitations to the use of Google Trends data. First, despite the evident potential that Google data have to offer in epidemiology and disease surveillance, there have been some issues in the past, where online search traffic data at some point failed to accurately predict disease spreading, as in the case of Google Flu Trends [57], a Google tool for the surveillance of influenza-like illness (the flu) that is no longer available. Regardless, Google Flu Trends has been accurate in the past in predicting the spread of flu, as suggested by several studies and reports [58-60].

The latter could be partly attributed to the fact that, when researching with Google Trends, the sample is unknown and it cannot be shown to be representative. Despite this and considering the increasing internet penetration, previous studies have suggested that Web-based data have been empirically shown to provide valuable and valid results in exploring and predicting behavior and are correlated with actual data [61-66]. However, recent research has suggested that online queries do not provide valid results in regions with low internet penetration or low scorings in freedom of speech [67].

Furthermore, the data that are retrieved are normalized over the selected period; thus, the exact volumes of queries are not known, limiting the way that the data can be processed and analysis can be performed. Therefore, the data should be analyzed in the appropriate way, and the results should be carefully interpreted.

In addition, the selection of keyword(s) plays a very important role in ensuring the validity of the results. In some cases, the noisy data (ie, queries not attributed to the examined term) must be excluded, which are not always trivial. This can be partly overcome by selecting a specific category, which always bares the risk of excluding results that are needed for analysis.

The analysis of Google Trends data has several other limitations, as examining Web data can bear threats to validity. Careful analysis should be performed to ensure that news reporting and sudden events do not compromise the validity of the results. In addition, as the sample is unknown, several other demographic factors such as age and sex cannot be included in the analysis.

Finally, as this field of research is relatively new, there is no standard way of reporting, resulting in the same meaning of different terms, different meanings of the same term, and different abbreviations. For example, Google Trends data are referred to as relative search volumes, search volumes, online queries, online search traffic data, normalized hits, and other terms. Thus, future research should focus on developing specific coding for Google Trends research, so that a unified way of reporting is followed by all researchers in the field.

In the era of big data, the analysis of Google queries has become a valuable tool for researchers to explore and predict human behavior, as it has been suggested that online data are correlated with actual health data. The methodology framework proposed in this article for researching with Google Trends is much needed to provide guidance for using Google Trends data in health assessment, and, more importantly, to help researchers and health officials and organizations avoid common mistakes that compromise the validity of the results. As research on the subject is expanding, future work should include the coding in Google Trends research and extend this framework along with changes in the tool and the analysis methods.

## Appendix

Multimedia Appendix 1 Google Trends categories.
