# Supplementary material for: Google Trends in Infodemiology and Infoveillance: Methodology Framework
Source: JMIR Public Health Surveill. 2019 May 29;5(2):e13439. doi: 10.2196/13439 (PMC6660120; doi:10.2196/13439)
Supplement: Multimedia Appendix 1 [file publichealth_v5i2e13439_app1.pdf]

Multimedia Appendix

Table A1. All levels of Available Categories and Subcategories of the "Health" Category

2nd level	3rd level	4th level
Ageing & Geriatrics ⇨	Alzheimer's Disease	
Alternative & Natural Medicine ⇨	Acupuncture & Chinese Medicine	
	Cleansing & Detoxification	
Health Conditions ⇨	AIDS & HIV	
	Allergies	
	Arthritis	
	Cancer	
	Cold & Flu	
	Diabetes	
	Ear, Noise & Throat	
	Eating Disorders	
	Endocrine Conditions ⇨	Diabetes<br>Thyroid Conditions
	Genetic Disorders	
	GERD & Digestive Disorders	
	Heart and Hypertension	
	Infectious Diseases ⇨	Cold & Flu<br>Parasites & Parasitic Diseases<br>Sexually Transmitted Diseases<br>Vaccines & Immunization
	Injury	
	Neurological Disorders ⇨	Alzheimer's Disease
	Obesity	
	Pain Management ⇩	Headaches & Migraines
	Respiratory Conditions ⇩	Asthma
	Skin Conditions	
	Sleep Disorders	
Health Education & Medical Training		
Health Foundations & Med. Research		
Health News ⇩	Health Policy	
Medical Devices & Equipment ⇩	Assistive Technology	
Medical Facilities & Services ⇩	Doctor's Offices	
	Hospitals & Treatment Centers	
	Medical Procedures	
	Physical Therapy	
Medical Literature & Resources ⇩	Medical Photos & Illustrations	
Men's Health ⇩	Erectile Dysfunction	
Mental Health ⇩	Anxiety & Stress	
	Depression	
	Learning & Development Disabilities ⇩	ADD & ADHD
Nursing ⇩	Assisted Living & Long Term Care	
Nutrition ⇩	Special & Restricted Diets ⇩	Cholesterol Issues
	Vitamins & Supplements	
Oral & Dental Care		
Pediatrics		
Pharmacy ⇩	Drugs & Medications	
Public Health ⇩	Health Policy	
	Occupational Health & Safety	
	Poisons & Overdoses	
	Vaccines & Immunisations	
Reproductive Health ⇩	Birth Control	
	Erectile Dysfunction	
	Infertility	
	OBGYN ⇩	Pregnancy & Maternity
	Sex Education & Counseling	
	Sexual Enhancement	
	Sexually Transmitted Diseases ⇩	AIDS & HIV
Substance Abuse ⇩	Drug & Alcohol Testing	
	Drug & Alcohol Treatment	
	Smoking & Smoking Cessation	
	Steroids & Performance-Enhancing Drugs	
Vision Care ⇩	Eye Glasses & Contacts	
Women's Health ⇩	OBGYN ⇩	Pregnancy & Maternity
